# Tau phosphorylation by GSK-3β promotes tangle-like filament morphology

**DOI:** 10.1186/1750-1326-2-12

**Published:** 2007-06-28

**Authors:** Carolyn A Rankin, Qian Sun, Truman C Gamblin

**Affiliations:** 1 Department of Molecular Biosciences, University of Kansas, Lawrence, KS, USA

## Abstract

**Background:**

Neurofibrillary tangles (NFTs) are intraneuronal aggregates associated with several neurodegenerative diseases including Alzheimer's disease. These abnormal accumulations are primarily comprised of fibrils of the microtubule-associated protein tau. During the progression of NFT formation, disperse and non-interacting tau fibrils become stable aggregates of tightly packed and intertwined filaments. Although the molecular mechanisms responsible for the conversion of disperse tau filaments into tangles of filaments are not known, it is believed that some of the associated changes in tau observed in Alzheimer's disease, such as phosphorylation, truncation, ubiquitination, glycosylation or nitration, may play a role.

**Results:**

We have investigated the effects of tau phosphorylation by glycogen synthase kinase-3β (GSK-3β) on tau filaments in an in vitro model system. We have found that phosphorylation by GSK-3β is sufficient to cause tau filaments to coalesce into tangle-like aggregates similar to those isolated from Alzheimer's disease brain.

**Conclusion:**

These results suggest that phosphorylation of tau by GSK-3β promotes formation of tangle-like filament morphology. The in vitro cell-free experiments described here provide a new model system to study mechanisms of NFT development. Although the severity of dementia has been found to correlate with the presence of NFTs, there is some question as to the identity of the neurotoxic agents involved. This model system will be beneficial in identifying intermediates or side reaction products that might be neurotoxic.

## Background

Tau is a remarkably soluble neuronal microtubule-associated protein that normally functions to promote the assembly and stabilization of the microtubule cytoskeleton. In Alzheimer's disease (AD) and other related neurodegenerative disorders, tau aggregates into straight and paired helical filaments [1]. As these diseases progress, the tau fibrils associate with one another to form large densely packed networks of interwined filaments termed neurofibrillary tangles (NFTs). Although the level of NFT formation has been shown to correlate with the severity of dementia [2,3], it is unclear whether NFT formation is neurotoxic. There are examples of NFT formation that correlate with neurodegeneration [4-6] and also examples of neurodegeneration in the absence of NFT formation [7-9]. It has even been suggested that NFT formation is protective for neurons [8]. However, NFTs are not inert pathological lesions, but rather follow a definite developmental progression (reviewed in [10]). It is possible that the neurotoxic agents in AD may be intermediates in the development of NFTs or even products of side reactions. Therefore, detailed studies of NFT formation would be beneficial to our understanding of toxic elements in AD.

Although polymerized tau is a major component of NFTs, numerous other molecules have also been found associated with NFTs. Some of these include ubiquitin [11,12], RNA [13], α-synuclein [14], GSK-3β [15], microtubule affinity regulating kinase [16], and apolipoprotein E [17]. One of these nonfibrillar components, GSK-3β, appears to have an active role in the pathological progression of neurodegeneration. A drosophila model expressing both human tau and the kinase GSK-3β exhibited enhanced neurodegeneration and neurofibrillary pathology [4] compared to expression of tau alone [9]. A similar result was seen in a transgenic mouse model expressing a mutated tau and the GSK-3β kinase [18]. To emphasize the role of phosphorylation, treatment with the GSK-3β inhibitor, lithium chloride, showed a reduction in neurodegeneration, tau phosphorylation, and tau pathology when administered at early stages of neuropathology [19,20]. Further evidence that phosphorylation may play a role in NFT development was the discovery of highly phosphorylated paired helical filaments, the form of filamentous tau most prevalent in AD (reviewed in [1,21,22]). Since GSK-3β appears to phosphorylate tau in many of the same phosphorylation sites identified in paired helical filaments of AD [23-25], it would appear that phosphorylation by GSK-3β may play a role in formation of fibrils or NFTs.

We have tested the hypothesis that GSK-3β is involved in some aspect of NFT formation by using an in vitro cell-free model, where reagents can be clearly defined and controlled. Cell-free polymerization models of tau have been well established, generally using either arachidonic acid (ARA) or heparin to initiate polymerization (reviewed in [26]). However, use of these models to study effects of tau phosphorylation on levels of tau polymer have been inconclusive [27-32]. We have found that phosphorylation of tau by GSK-3β had little effect on polymerization levels, although it had a significant role in the formation of large localized accumulations of intertwined filaments. These aggregates of tau fibrils were stable through sucrose gradient centrifugation and migrated to the same region as NFT-like structures isolated from AD brain [33,34]. Further investigation of the reaction conditions producing these NFT-like structures showed that the concentration and nature of inducer(s) were important factors in defining aggregation characteristics of size and density. This cell-free model of the formation of NFT-like accumulations provides a useful tool for future studies to understand how aggregation of tau polymer into NFT-like structures might occur and which steps in the process might potentially produce neurotoxic products.

## Results

### Phosphorylation of monomeric tau by GSK-3β

**Figure 1:**
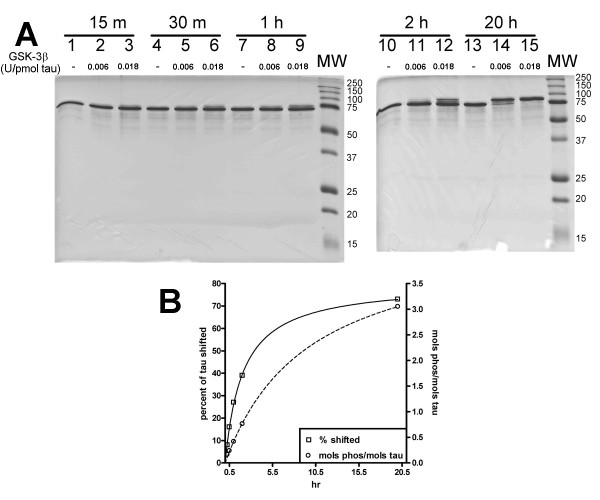
**Tau phosphorylation by GSK-3B**. A) SDS-PAGE analysis of tau protein incubated for 20 h in the absence (lanes 1, 4, 7, 10, and 13) or the presence of 0.006 U GSK-3β per pmol tau (lanes 2, 5, 8, 11, 14) or 0.018 U GSK-3β per pmol tau (lanes 3, 6, 9, 12, and 15). A definite band shift in the migration of phosphorylated tau can be detected with increasing time and kinase concentration. B) The amount of γ-^32^P incorporation over time using 0.018 U GSK-3β per pmol tau (open circles, right y-axis) is compared to the SDS-PAGE analysis in Panel A (open squares, left y-axis). Lines are drawn through the points to ease comparison. Data represents a single trial.

The in vitro phosphorylation of monomeric tau by GSK-3β was dependent on both enzyme concentration and incubation time (Figure 1A). Phosphorylation of tau was detected by an upward shift in mobility upon SDS-PAGE analysis reminiscent of hyper-phosphorylated tau in AD [22]. The band shift represents an SDS-resistant conformational change brought about by phosphorylation rather than a molecular weight increase due to added phosphates [35,36]. The in vitro phosphorylation, seen as an upward band shift, was mostly complete after 20 h incubation (Figure 1A, lanes 13, 14, and 15). With no GSK-3β present (lane 13) the tau monomer migrates approximately as a 74 kDa protein. With a kinase concentration of 0.006 U/pmol tau (lane 14), the presence of multiple bands suggest that tau is not fully phosphorylated. At a kinase concentration of 0.018 U/pmol tau (lanes 3, 6, 9, 12, and 15), band density measurements showed that 8% shifted after 15 minutes, 16% by 30 min, 29% by one hour, 39% by 2 hours and 73% after 20 hours.

The number of phosphates incorporated per mole of tau was quantified by utilizing [γ-^32^P] ATP as a substrate (Figure 1B). In comparing gel shift and radioactive phosphate incorporation data (Figure 1B) a similar increase in phosphorylation over time was observed. At 20 h incubation and a GSK-3β concentration of 0.018 U/pmol tau, approximately 3 moles of phosphate were incorporated per mole of tau, which is similar to the 2–4 mols phosphate incorporated/mol of tau in previously published reports of in vitro GSK-3β phosphorylation of tau [37].

### Sites phosphorylated by GSK-3β in tau monomer

**Figure 2:**
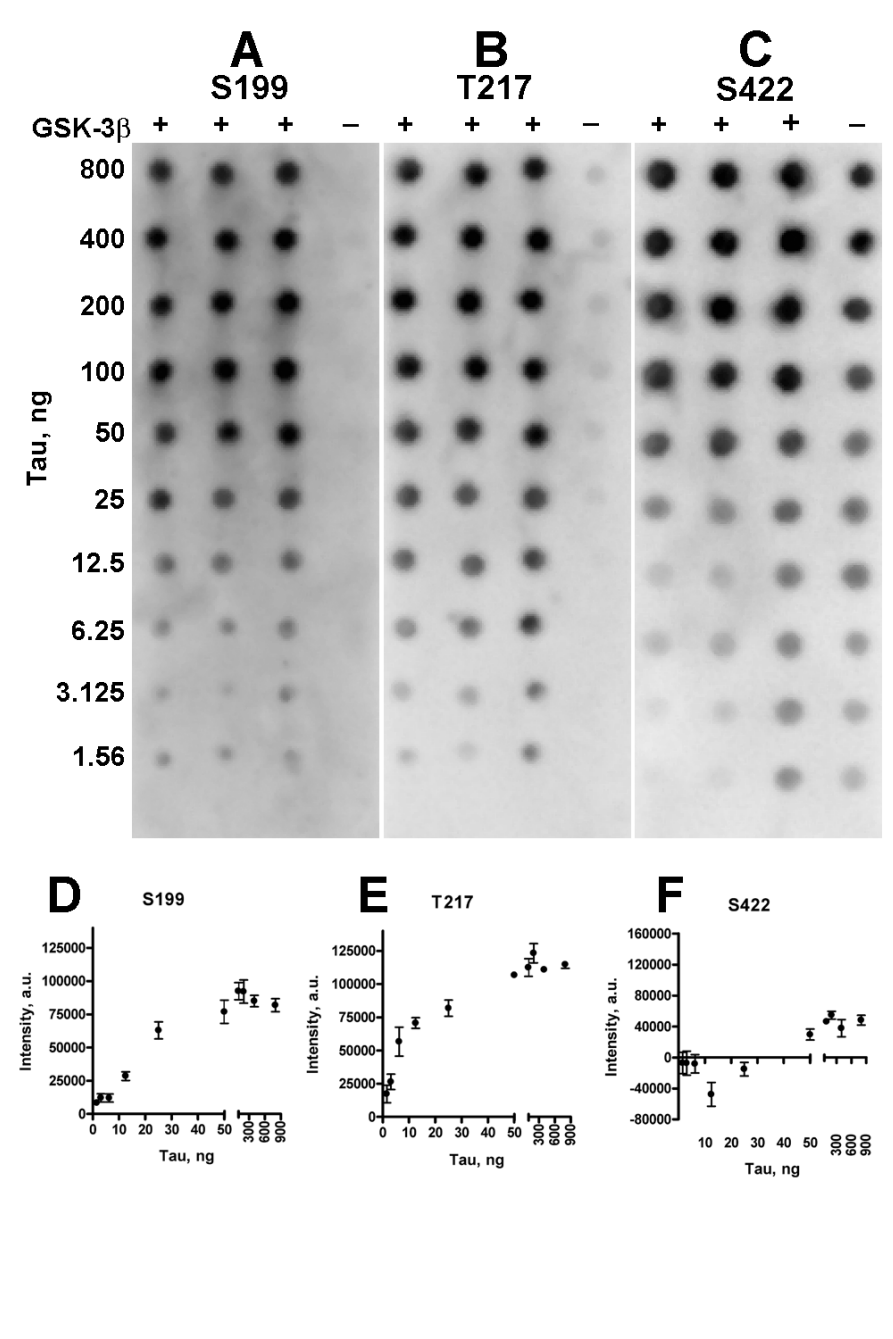
**Tau sites phosphorylated by GSK-3β in an in vitro reaction**. Thirteen phosphorylation-site-specific antibodies were used to probe dot blots of GSK-3β phosphorylated tau. Panel A and B show two representative blots. Three repetitions of each phosphorylated tau concentration, ranging from 800 to 1.56 ng, were spotted on a blot, then probed with an anti-phosphorylation specific antibody (See Materials and Methods for details). To estimate non-specific background levels, each blot also included one spot of non-phosphorylated tau at each concentration. Following density analysis, the antibody titrations were plotted (Panels D and E) and 1/2 max concentrations were estimated. Anti-S422 antibody shown in Panel C, and its titration plot in Panel F, did not recognize phosphorylation and served as a negative control.

We determined which tau sites were phosphorylated by utilizing a panel of phosphorylation specific antibodies. Thirteen dot blots containing concentrations of phosphorylated tau ranging from 1.56 to 800 ng per dot were probed with these antibodies. Representative dot blots are shown in Figure 2. Non-specific background binding to non-phosphorylated tau was subtracted and titration curves were plotted and analyzed (see Materials and Methods). The blot probed with anti-S199 showed very little non-specific antibody binding to non-phosphorylated tau (Figure 2A); anti-T217 showed slightly more non-specific binding (Figure 2B); and anti-S422 was the least specific antibody under these conditions (Figure 2C). The titration curves of most of the antibodies resembled that of anti-S199 (Figure 2D). These included anti-T205, -S214, -T231, -S262, -S356, -S400, -S404, and -S409. Anti-T212 and -S396 titration curves more closely resembled that of anti-T217 (Figure 2E). Only anti-tau S422 did not recognize a specific phosphorylation site (Figure 2C, F) and therefore, served as a negative control. The 1/2 max of most antibodies was in the range of 20–40 ng; only T212 differed, with a 1/2 max of roughly 12 ng. Our phosphorylation specific antibody data was in agreement with those sites recognized by the mass spectrometry data of Reynolds, et al. [37] and the combination of two dimensional phosphopeptide mapping and mass spectrometry used by Connell, et al. [38], except that we identified additional sites, T205, S214, S262, S356, and S409 that were not identified by mass spectrometry (Table 1). We did not have an antibody to the S235 site which was identified in both the Connell, et al. and Reynolds, et al. studies [37,38].

**Table 1:** Tau sites phosphorylated by GSK-3β.

sites	Reynolds^a^	Connell^b^	Rankin^c^
T175	+		*
T181	+		*
S199	+	+	+
T205			+
T212	+	+	+
S214			+
T217	+	+	+
T231	+	+	+
S235	+	+	*
S262			+
S356			+
S396	+	+	+
S400	+	+	+
S404	+	+	+
S409			+
S422			

Identification of in vitro phosphorylated sites by mass spectrometry

^a ^Reynolds, et al. study [37]

^b ^Connell, et al. study [38]

^c ^Identification of in vitro phosphorylated sites by phosphorylation site-specific antibodies. Antibodies were unavailable for those sites marked with an asterisk. S422, not identified by either mass spectrometry or antibody analysis, was a negative control.

### Kinetic analysis comparing polymerization of GSK-3β phosphorylated tau with polymerization of non-phosphorylated tau

**Figure 3:**
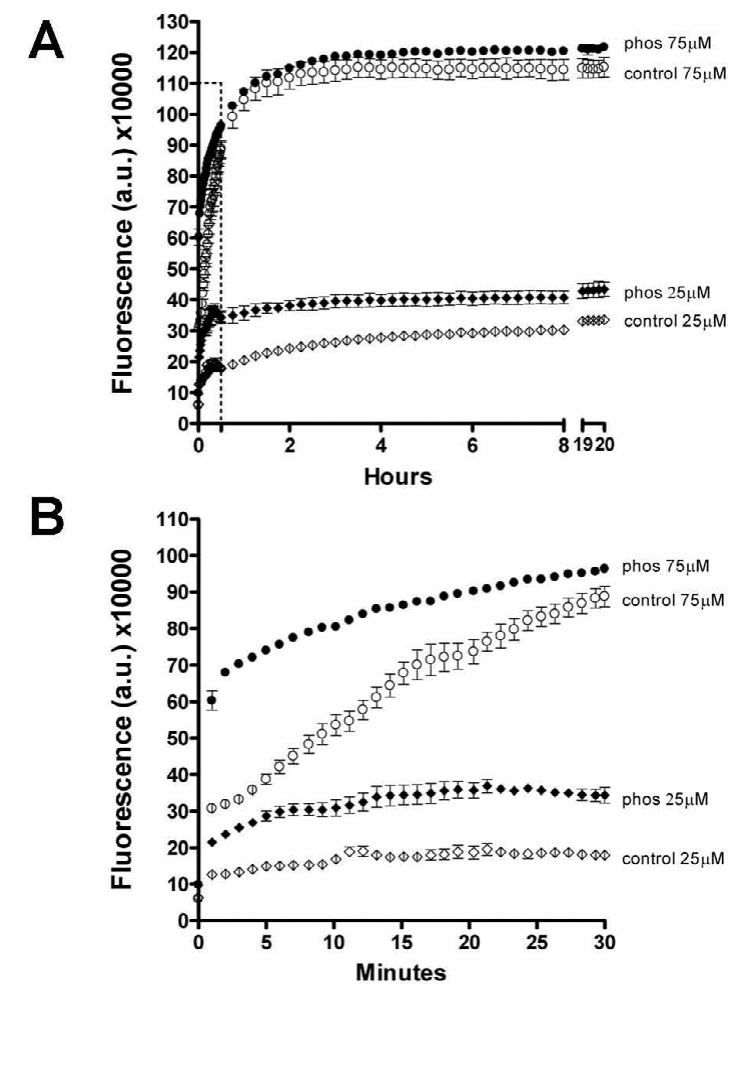
**Kinetic analysis of polymerization using GSK-3β phosphorylated tau**. A comparison between control (non-phosphorylated, open symbols) and phosphorylated tau (filled symbols) showed that phosphorylation altered polymerization kinetics, particularly in the first 30 minutes of the reaction. Panel A shows kinetics over the entire 20-h reaction time. Panel B shows only the first 30 minutes (the section boxed by a dotted line in panel A). Two ratios of ARA inducer:tau protein were compared: a suboptimal ratio (labeled 25 μM, representing the concentration of ARA, diamonds) and an optimal ratio (labeled 75 μM, circles). The tau protein concentration was 2 μM for all kinetic reactions. Changes in ThS fluorescence intensity (y axis) was used to indicate the extent of polymerization and measurements were in arbitrary units (a.u.). Error bars are +/- SEM. Every 40th data point was plotted for ease in interpretation.

Thioflavine S (ThS) fluorescence intensity, a method traditionally used to obtain kinetic information regarding tau filament formation in vitro [39] was used to compare polymerization kinetics (Figure 3). Tau polymerization reactions are described in detail in Materials and Methods. The polymerization reactions were performed with optimal or suboptimal ratios of ARA inducer to tau protein concentrations. For polymerization purposes, an optimal ratio of ARA inducer to tau is 75 μM ARA:2 μM tau; a suboptimal ratio is 25 μM ARA:2 μM tau [40]. The reaction kinetics represented by an increase in ThS fluorescence intensity were monitored for 20 h. At the optimal ratio of ARA inducer to tau protein (labeled 75 μM), the slight increase in polymerization of GSK-3β phosphorylated tau observed at the end of the reaction was not significant (Figure 3A) (P = 0.1788). However, at a suboptimal ratio (labeled 25 μM) phosphorylated tau had a significant increase in polymerization over non-phosphorylated control (Figure 3A) (P = 0.0142).

Assaying by ThS intensity allows for observation of changes very early in the reaction and upon the addition of ARA inducer there was a rapid and dramatic increase in ThS fluorescence (Figure 3B), as has been previously described [41]. This rapid early increase in ThS intensity apparently registers a change in molecular structure but does not measure filament formation per se [41]. At both suboptimal and optimal inducer:protein ratios, the phosphorylated protein seemed to have a greater initial velocity of polymerization compared to non-phosphorylated tau (Figure 3B), suggesting that phosphorylated protein is either in an altered conformation or more rapidly adopts a ThS positive conformation in the presence of ARA.

### TEM analysis of filaments from GSK-3β phosphorylated tau

**Figure 4:**
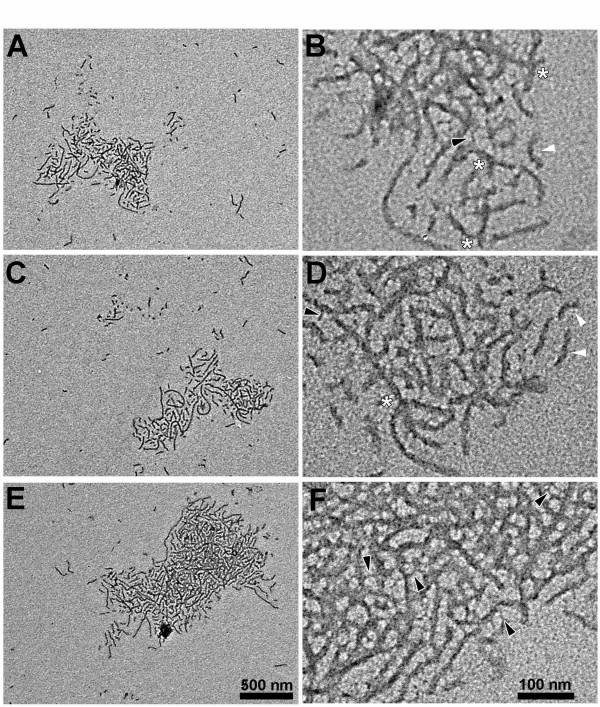
**Clustering of filaments polymerized from phosphorylated tau**. Samples from the 25 μM ARA-induced phosphorylated tau reaction (20 h) shown in Figure 3, were diluted five-fold and prepared for transmission electron microscopy (TEM). Panels A, C and E show representative clusters of filaments at a magnification of 50,000 × (scale bar represents 500 nm). Panels B, D and F show the same clusters at a magnification of 100,000 × (scale bar represents 100 nm). In the higher magnification micrographs, white arrowheads denote non-touching filaments; white asterisks, branching filaments; and black arrowheads, tendril-like fibrils that appear to laterally connect larger adjacent filaments.

**Figure 5:**
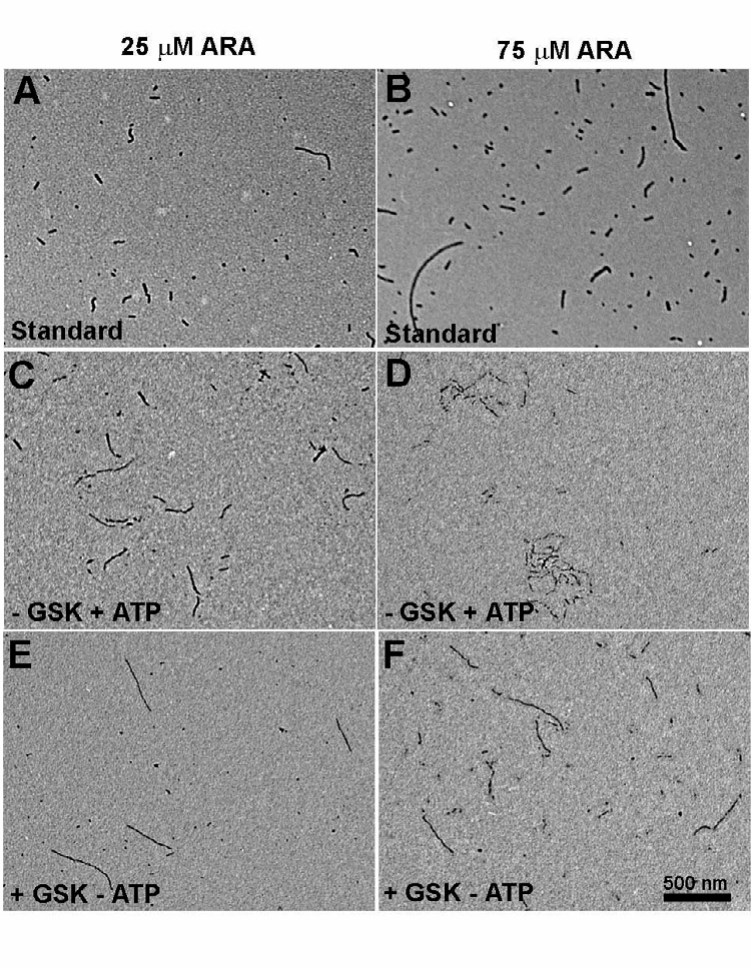
**TEM analysis of mock phosphorylation reactions**. Standard polymerization reactions containing 2 μM tau were induced with either a low (25 μM), or optimal (75 μM) concentration of ARA (Panels A and B, respectively). Panels C-F show polymerization reaction products in which mock-phosphorylated tau had been polymerized. In panel C and D, GSK-3β had been omitted from the phosphorylation reaction (- GSK + ATP) and in panel E and F, ATP had been omitted from the phosphorylation reaction (+ GSK – ATP). The 25 μM ARA reactions were diluted five fold, the 75 μM ARA reactions were diluted ten fold prior to grid preparation.

Polymerization samples of phosphorylated tau (induced with 25 μM ARA) were taken from the 20 h time point in the kinetic analysis described above and prepared for TEM. The filaments produced in the GSK-3β phosphorylated tau reactions were not distributed uniformly on the TEM grid, but clustered together into discrete aggregates (Figure 4A, C, and 4E). At higher magnifications (Figure 4B, D, and 4F), some filaments, although appearing to be a part of the aggregate, were not touching (Figure 4, white arrowheads); others seemed to be intertwined. Additional configurations appeared to represent branching filaments (Figure 4, white asterisk) as well as tendril-like fibers bridging the lateral gap between larger adjacent filaments (Figure 4, black arrowheads). The filaments within the aggregates had an average width of 16 ± 4 nm, similar to non-aggregated filaments seen in Figure 4, and to filaments formed from non-phosphorylated tau (Figure 5A and 5B).

The major advantage of an in vitro cell-free system as a research tool is the ease with which various reagents (by their addition/omission) can be examined for their effect on a specific result, in this case the clustering or aggregation of filaments. Conventional, control reactions with non-phosphorylated tau, performed under standard polymerization conditions with either the suboptimal or optimal ratios of ARA:tau protein (75 or 25 μM ARA, respectively:2 μM tau protein) did not show filament clustering (Figure 5A and 5B). Polymerization reaction conditions are detailed in Materials and Methods. Since the 75 μM ARA TEM sample was diluted by a factor of ten whereas the 25 μM ARA sample was diluted by a factor of five, the TEM analysis appeared similar to the kinetic study observations (Figure 3) in regard to filament mass.

Since GSK-3β is a component of NFTs in AD, it was possible that its primary effect on cluster formation came not from its role as a kinase but from a hypothetical role as NFT or cluster "glue". To determine the role of GSK-3β, we carried out two mock phosphorylation reactions, one in which the GSK-3β was omitted (Figure 5C, D), the other in which the ATP was omitted (Figure 5E, F). Omission of ATP mimics the GSK-3β inhibitors that act by competing for ATP. Without ATP in the phosphorylation reaction, no filament clustering occurred in the polymerization reaction (Figure 5E, F) and none of the mock phosphorylation reactions showed a band shift on SDS-PAGE (data not shown). Since GSK-3β did not support filament clustering without ATP it would seem that phosphorylation of tau is the primary role of GSK-3β in cluster formation. Although polymerization of the mock-phosphorylated tau induced by 25 μM ARA showed no filament clustering if either GSK-3β or ATP were omitted (Figure 5C and 5E), at high ARA concentration (75 μM) we did observe a few small filament clusters when ATP was present without GSK-3β, suggesting that high inducer concentration plus ATP might partially compensate for tau phosphorylation in cluster formation.

**Figure 6:**
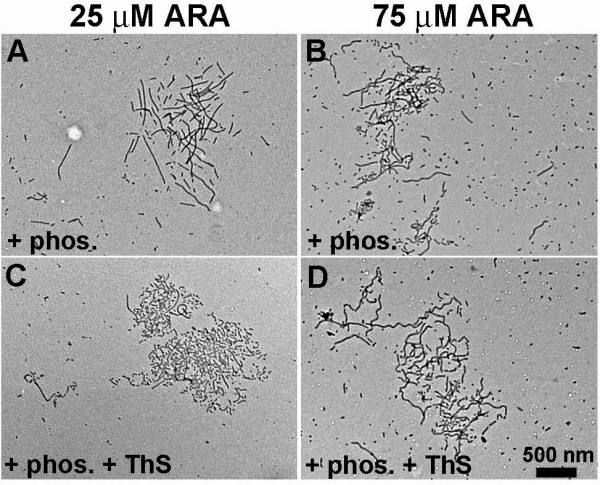
**Phosphorylation by GSK-3β is sufficient to promote filament clustering**. Panel A and B show products of phosphorylated and polymerized tau (+phos.). Panel C and D show products of phosphorylated tau that was polymerized in the presence of 20 μM ThS (+phos., +ThS). The clusters of filaments were approximately 2 μm across their longest axis. Magnification was 20,000 × (scale bar represents 500 nm). Panels A and C were induced with 25 μM ARA and diluted five fold prior to grid preparation; Panels B and D were induced with 75 μM ARA, and diluted 10 fold.

To determine whether phosphorylation by GSK-3β alone was sufficient to promote tau filament clustering, phosphorylated tau was polymerized without the ThS present in the samples from the kinetic study. This was important since high concentrations of ThS (100 μM) have been shown to induce polymerization of monomeric tau [42] and possibly, a lower concentration of ThS was required in addition to phosphorylation to promote cluster formation. TEM analysis showed that phosphorylation alone was sufficient to promote cluster formation with either ratio, 25 or 75 μM ARA:2 μM tau (Figure 6A, B). However, especially with the suboptimal inducer:tau ratio (25 μM ARA), the cluster morphology appeared to change with the addition of ThS (Figure 6, compare A with C). None of the clusters formed with the optimal ARA:tau ratio (75 μM ARA) were as tightly packed as those with 25 μM ARA and 20 μM ThS (Figure 6, compare B and D with C). These apparent differences in cluster morphology suggested that inducer concentration and/or ThS might be cluster modifiers.

### Morphological characteristics of filament clusters

To verify our TEM observations (and to assess the statistical significance of potential modifying reagents), the number of filaments and the average filament length in each cluster, as well as the area covered by each cluster were measured. These cluster properties, average filament length, filament number, area covered, and density allowed us to quantify cluster morphology and assess the modifying effects of various reagents.

**Figure 7:**
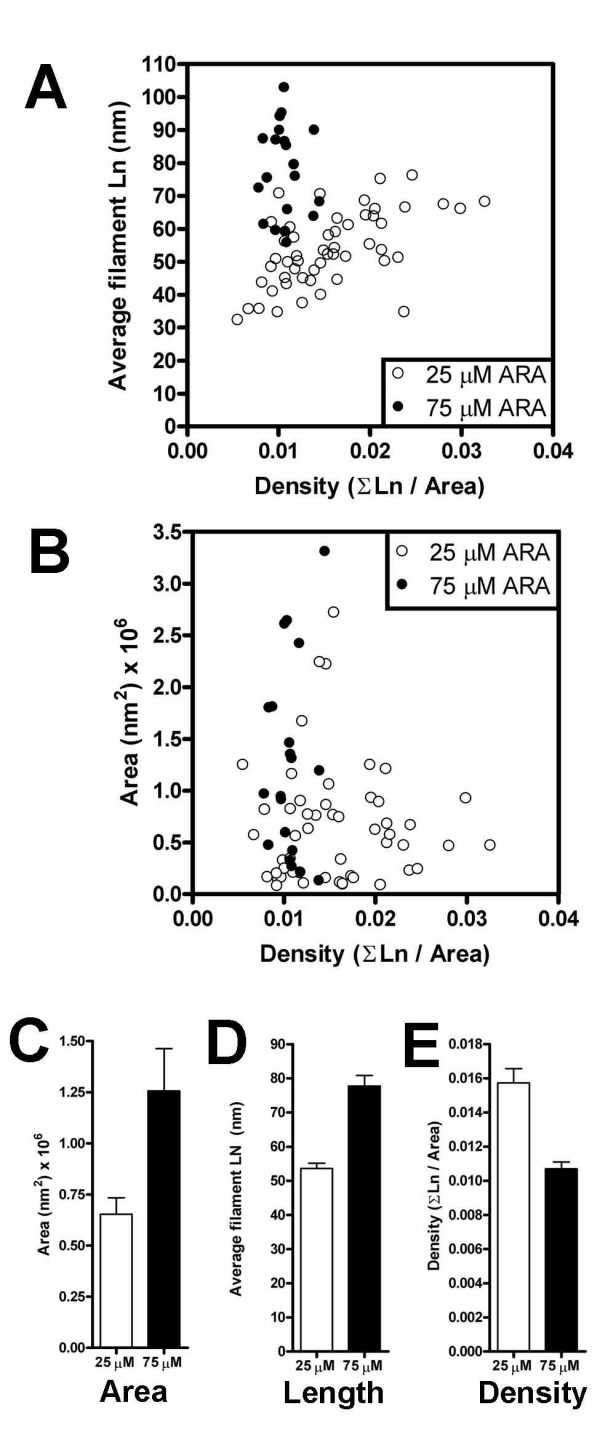
**Morphological characteristics of clusters**. The average length of filaments in a cluster (Panel A) and the area covered by each cluster (size) (Panel B) were determined and plotted against the calculated density of the cluster. Each cluster is represented separately in Panels A and B. This data was then averaged and summarized in Panel C (area), Panel D (filament length) and Panel E (density). Polymerization products from 25 μM ARA-induced polymerization reactions are presented as white circles and bars; those from 75 μM ARA induction are presented as black circles and bars. Error bars are +/- SEM.

We first compared clusters formed by polymerizations of phosphorylated tau plus ThS (+phos.+ThS) that were induced with 25 μM ARA to those induced with 75 μM ARA (Figure 7). The comparison showed that the average length of the filaments within each cluster was significantly increased with the optimal (75 μM) ARA concentration (Figure 7A, D) (P < 0.0001), as was the area covered by each cluster (Figure 7B, C) (P = 0.0148). However, the clusters were less densely packed than those formed with 25 μM ARA (Figure 7E) (P = 0.0014). These properties are summarized in Figure 7C, D, and 7E, and are in agreement with the representative TEM micrographs of Figure 6C and 6D. The ratio of inducer:tau in the polymerization reaction apparently affects one or more of these properties of cluster morphology and determines how the filaments interact to affect size and density of the clusters. These measurements demonstrate that cluster morphology can be defined by quantitative measurements and that the morphology can be modified by specific reaction conditions.

### Effect of ThS on filament formation

**Figure 8:**
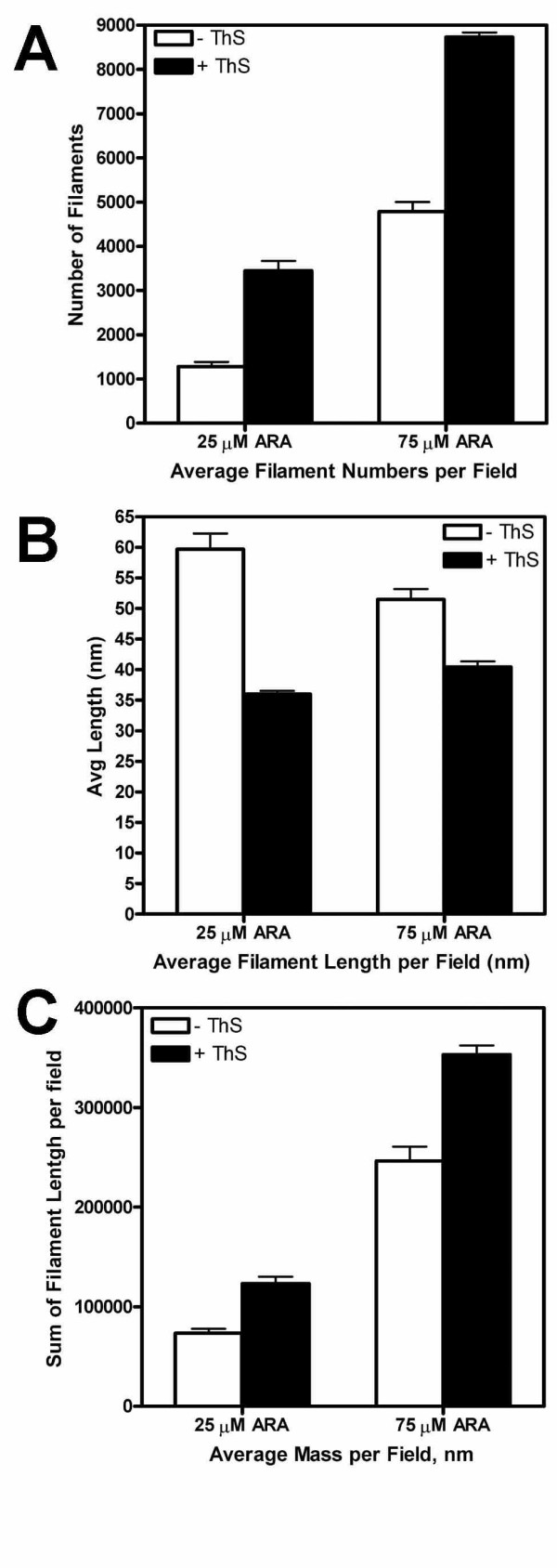
**Effects of ThS on filament formation**. Polymerization reactions were carried out with 2 μM non-phosphorylated tau under standard conditions with low (25 μM) or optimal (75 μM) ARA inducer:tau ratios without (white bars) or with (black bars) ThS. Error bars represent the SEM. Panel A is a comparison of the number of filaments; Panel B is a comparison of average length of filaments; and Panel C is the calculation of filament "mass" (detailed in Materials and Methods).

TEM micrographs suggested that ThS might also modify cluster morphology (Figure 6). To isolate the effect of ThS on filament formation, we examined wild type, non-phosphorylated tau polymerization reactions with and without ThS (Figure 8A–C). The presence of ThS resulted in a significant increase in the number of filaments (Figure 8A), a significant decrease in average filament length (Figure 8B), and a significant increase in the calculated mass of filaments per field (Figure 8C) for both 25 μM and 75 μM inducer concentrations (all P values were <0.0001). This data suggests that conditions which produce shorter filaments promote formation of a tighter and more densely packed cluster.

### Comparison of filament clusters formed in vitro to NFT-like filament bundles isolated from AD brain

**Figure 9:**
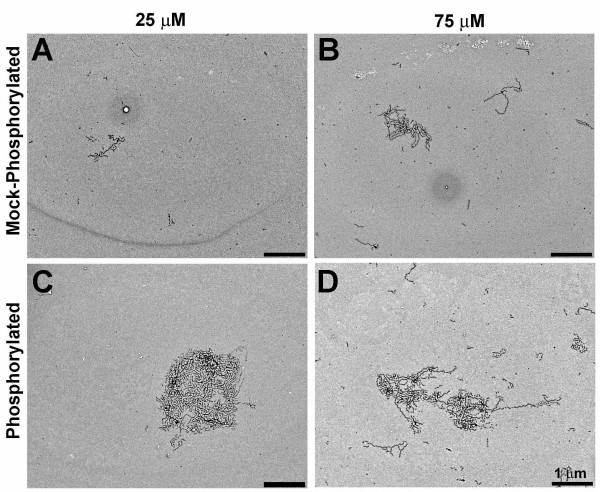
**Sucrose gradient centrifugation of polymerization reactions**. Polymerization reactions containing ARA, ThS and either mock-phosphorylated (minus GSK-3β), or phosphorylated tau were centrifuged through a discontinuous sucrose gradient containing 1.0, 1.5, and 2.0 M sucrose. Filament clusters sedimented at the interface of the 1.5 and 2.0 M sucrose concentrations. Panels A, and C are TEM micrographs of gradient fractions from reactions induced with a low inducer:tau ratio (25 μM ARA:2 μM tau, panels B and D with an optimal ratio (75 μM ARA:2 μM tau). Grids were prepared without glutaraldehyde fixation of polymerization samples. Samples were concentrated five fold on the TEM grids.

In order to compare the similarity of the clustered filaments in our in vitro cell-free system to isolated NFT-like bundles of tau filaments from AD brain, polymerization reaction products were fractionated using centrifugation through a discontinuous gradient of 1.0, 1.5 and 2.0 M sucrose in polymerization buffer. The procedure was based on a published protocol for separating individual tau filaments from NFT-like bundles of tau filaments isolated from AD brain [33,34]. Following centrifugation, numerous clusters of phosphorylated filaments accumulated at the 1.5/2.0 sucrose interface (Figure 9C and 9D), the same place that the larger NFT-like bundles of tau filaments from human AD brain had accumulated [33]. A few very small clusters of mock-phosphorylated tau filaments were also found (Figure 9A, B). Since we previously found that mock phosphorylation conditions containing ATP in the absence of GSK-3β did produce a small number of tangles at a high ARA:tau ratio (Figure 5D), it is conceivable that tangles might also form, albeit more rarely, with the suboptimal ratio (25 μM ARA). However, due to the rare occurrence of tangles under these conditions, concentration by the sucrose gradient was required for detection. Since proteins migrate in density-gradient (zonal) centrifugation according to weight, density and shape, the sucrose gradient experiment suggests that the clustered filaments formed in vitro and the larger aggregates isolated from AD brain share similar properties. Furthermore, the in vitro filament aggregates are quite stable, maintaining their distinctive morphology through sucrose gradient centrifugation and extensive washing prior to examination by TEM.

## Discussion

Due to the controversial role of neurofibrillary tangle (NFT) formation in the neurodegenerative process [2-4,6-9,43-47], a better understanding of the mechanisms leading to the formation of NFT would be beneficial to our understanding of AD.

In this report, we have demonstrated that GSK-3β phosphorylation of tau is sufficient to induce the clustering of ARA-induced filaments into structures similar to the NFT-like aggregates of tau filaments purified from AD brain [33,34]. These results suggest that GSK-3β phosphorylation not only produces a small but significant increase in tau filament formation, but also shows that phosphorylation alters the nature of interactions between those filaments resulting in their clustering into NFT-like structures. Although in this report we address only the effects of tau phosphorylation by GSK-3β, ARA inducer concentration (the inducer:tau ratio in polymerization), and ThS on the clustering of tau filaments into NFT-like structures, we feel that this is an important first step in unraveling the molecular mechanisms of NFT formation through cell-free in vitro modeling.

The clusters of tau filaments formed by polymerization of GSK-3β phosphorylated tau are stable and their formation is readily reproducible, although various factors influence the size, mass and density of the clusters. Here we demonstrate the effects of inducer and the ratio of inducer:tau concentration on these properties. In general, we have found that phosphorylation by GSK-3β is sufficient for cluster formation. In addition, conditions that alter filament length modify both the density of the filaments in the cluster, and the size of the cluster. Increases in inducer concentration which result in a change from the suboptimal to optimal ratio of inducer:tau concentration in the polymerization reaction increase the filament length within clusters and the area covered by the clusters. This results in clustered filaments that are less densely packed. Conversely, conditions that decrease filament length produce smaller clusters that contain a higher density of filaments.

With this newly developed in vitro model, we can begin to dissect the molecular mechanisms that are involved in filament aggregations that form NFT-like structures. Further studies will be aimed at understanding whether GSK-3β phosphorylation unmasks regions of tau molecules that interact with one another in forming clusters or whether the GSK-3β phosphorylation sites are interacting directly. It is tempting to speculate a role for the former since GSK-3β phosphorylation results in an SDS-resistant conformational change as observed by an upward shift in mobility on SDS page analysis. The apparent increase in initial polymerization velocity as monitored by ThS fluorescence also suggests that the GSK-3β phosphorylated tau may be in a conformation that more readily interacts with the ARA inducer or with the ThS as used to detect amyloid-type interactions in tau kinetic analyses. Although the co-localization of GSK-3β with tau pathology in AD suggests that NFTs may form from the direct interaction of GSK-3β with tau filaments [15], our mock-phosphorylation results strongly suggest that phosphorylation is the primary role of GSK-3β in promoting cluster formation.

This in vitro model for NFT formation requires the induction of tau polymerization via the addition of ARA, which may lead to the questioning of its physiological relevance. An inducer of tau polymerization in AD and other neurodegenerative disorders has not been identified, but that does not dampen our enthusiasm for the use of ARA as an inducer in our cell-free in vitro model system. This is due to ample evidence that ARA induced tau filaments are structurally similar to filaments from AD [42,48-51]. Additionally, there is growing evidence that ARA or its metabolites could be involved in the neurodegenerative process in AD (reviewed in [52]). While a direct connection between tau polymerization and ARA remains to be made in AD, the structural similarity between ARA induced filaments and AD filaments, plus the similarity between GSK-3β induced NFT-like clusters of tau filaments and those found in AD provide a strong argument for the physiological relevance of this model. In addition to the characterization of the GSK-3β induced clustering of filaments, this in vitro model provides a tool for investigating whether other kinases such as cyclin dependent kinase 5 or microtubule affinity regulating kinase have similar properties to induce the formation of NFT-like filament bundles. Likewise, other modifications found in association with AD NFTs, such as truncation, ubiquitination, nitration and glycation (reviewed in [1,10]) could also be tested. Our hope is that these ongoing studies will isolate factors contributing not only to the formation of NFT-like clusters, but also to identify the conditions that could lead to potentially toxic tau aggregates in cell and animal culture models.

## Methods

### Reagents and Supplies

Arachidonic acid was obtained from Cayman Chemicals (Ann Arbor, MI), thioflavine S and recombinant glycogen synthase kinase 3β (GSK-3β) from Sigma (St. Louis, MO), and uranyl acetate and formvar carbon coated grids from Electron Microscopy Sciences (Hatfield, PA). SDS-PAGE markers are Precision Plus Protein Standards from Bio-Rad (Hercules, CA).

### Tau protein

Tau protein (441 amino acids containing exons 2, 3 and 10) was expressed in and purified from BL21 E. Coli as described previously [32]. Protein concentration was determined using the Pierce BCA assay (Pierce Biotechnology, Rockford, IL). The purity of the protein was assessed by SDS-PAGE electrophoresis.

### Phosphorylation of tau by GSK-3β

#### Determination of optimal phosphate incorporation

Tau protein at a final concentration of 16 μM was incubated with either 0.006 or 0.018 U GSK-3β per pmol tau in buffer containing 40 mM HEPES, pH 7.64, 5 mM EGTA, 3 mM MgCl_2_, and 2 mM ATP for 20 h at 30°C. One unit of GSK-3β is defined as the amount of enzyme that will transfer one pmol phosphate from ATP to phosphatase inhibitor 2 per min at pH 7.5 at 30°C. Samples were removed at various times from the phosphorylation reaction, then boiled in Laemmli sample buffer [53] for 5 minutes to stop phosphorylation. One microgram of tau protein from the reaction time points was analyzed by SDS-PAGE.

#### Determination of gel band shift

SDS-PAGE gels from posphorylation reactions were converted to digital images using an HP Scanjet 7400c scanner (Hewlett-Packard company, Palo Alto, CA). The gels were converted to grayscale and inverted using Adobe Photoshop (Adobe Systems Incorporated, San Jose, CA). Two fixed size marquees were used to determine the average intensity of pixels from the entire sample in a lane or only the area corresponding to the shifted band. The average intensity of the gel background was subtracted. The percentage of tau in the shifted band was determined by dividing the value for the shifted band by the value for the total tau in the lane.

#### Determination of phosphate incorporation

Tau protein at a final concentration of 16 μM was incubated with 0.018 U GSK-3β per pmol tau in buffer containing 40 mM HEPES, pH 7.64, 5 mM EGTA, 3 mM MgCl_2_, and 2 mM ATP containing 10 μCi [γ-^32^P] labeled ATP (Specific activity: 3000 Ci/mmol) (Perkin-Elmer, Boston, MA) for 20 h at 30°C. Samples were removed at various times from the phosphorylation reaction, diluted and filtered through a Millipore ULTRAFREE 10,000 molecular weight cut off filter (Millipore, Billerica, MA). Samples were washed with two-250 μl volumes buffer containing 5 mM DTT, 100 mM NaCl, 10 mM HEPES pH 7.64, and 0.1 mM EDTA. γ-^32^P incorporation in tau was measured using a Packard 1600TR liquid scintillation counter to measure the radioactivity in the retentate and filter. The protein content of the flow-through filtrate was assayed by the BCA microplate protocol (Pierce Biotechnology, Rockford, IL) and was below the minimum detectable amount (20 μg/ml), allowing the assumption that no protein was lost during the filtration process. The amount of phosphate per protein molecule was calculated using the specific activity of γ-^32^P and the molar concentration of tau.

#### Generation of GSK-3β phosphorylated tau for further analysis

Based on the results above, we determined that the optimal phosphate incorporation was achieved by incubating 16 μM tau with 0.018 U GSK-3β per pmol of tau in buffer containing 40 mM HEPES, pH 7.64, 5 mM EGTA, 3 mM MgCl_2_, and 2 mM ATP for 20 h at 30°C. These conditions were used to generate GSK-3β phosphorylated tau for use in Figures 2, 3, 4, 6, 7, and 9.

#### Mock-phosphorylated tau

Two separate conditions were used to generate "mock" phosphorylated tau analyzed in Figure 5. The first was by eliminating GSK-3β from the reaction such that the phosphorylation reaction consisted of 16 μM tau in buffer containing 40 mM HEPES, pH 7.64, 5 mM EGTA, 3 mM MgCl_2_, and 2 mM ATP for 20 h at 30°C and was also used as a control in Figure 9. The second was by eliminating ATP from the reaction such that the phosphorylation reaction consisted of 16 μM tau with 0.018 U GSK-3β per pmol of tau in buffer containing 40 mM HEPES, pH 7.64, 5 mM EGTA, and 3 mM MgCl_2_, and then incubated for 20 h at 30°C.

### Dot blots of phosphorylated tau protein

Phosphorylated tau was diluted in TBS (20 mM Tris pH 7.5, 150 mM NaCl) such that 3 μl of the dilution contained the desired amount of protein. The protein (3 μl) was spotted onto Immobilon P membrane which was prepared according to manufacturer's instructions (Millipore, Billerica, MA). Blots were blocked for 1 h in TBS containing 1% BSA and 2% normal goat serum. The primary, tau phosphorylation-specific antibodies (Biosource International, Camarillo, CA) were diluted 1:1000 in blocking solution and blots were rotated overnight at 4°C. Blots were washed with TBS/Tween 20 (0.1%)/NP40 (0.05%), blocked with blocking buffer and incubated with an alkaline phosphatase conjugated anti-rabbit IgG secondary antibody (Sigma, St. Louis, MO). Blots were developed with chemiluminescence reagent, CDP-Star (PerkinElmer Life Sciences, Boston, MA). Images were captured on a Kodak Image Station 4000R (Eastman Kodak Co, Molecular Imaging Systems, Rochester, NY), and analyzed using the Array Analysis feature of the ImageQuantTL v2003.03 software that accompanies the Typhoon Trio, Variable Mode Imager (Amersham Biosciences, Piscataway, NJ). After subtracting non-specific binding data (density of the non-phosphorylated tau dots for each concentration), the density data was plotted using GraphPad Prism 4 GraphPad Software Inc., San Diego, CA).

### Tau polymerization reactions

#### Standard polymerization reactions

Tau was diluted to a final concentration of 2 μM into buffer containing 10 mM HEPES, pH 7.64, 100 mM NaCl, 0.1 mM EDTA, and 5 mM DTT. Arachidonic acid (ARA) was added to a final concentration of either 25 or 75 μM to induce polymerization. Standard polymerization reaction conditions were used as controls for Figures 3, 5 and 8.

#### Polymerization of phosphorylated tau

In polymerization reactions using phosphorylated tau, the phosphorylation reaction was diluted to a final concentration of 2 μM into polymerization buffer containing 10 mM HEPES, pH 7.64, 100 mM NaCl, 0.1 mM EDTA, and 5 mM DTT. This resulted in some minor buffer additions: 0.625 mM EGTA, 0.375 mM MgCl2, 0.25 mM ATP and increased the final HEPES concentration to 15 mM. These buffer additions did not appear to affect polymerization. The GSK-3β that carried over from the phosphorylation reaction was not de-activated. Phosphorylation reaction products were analyzed by coomassie blue staining on 10–15% SDS-PAGE prior to use in polymerization reactions.

#### Polymerization of mock phosphorylated tau

The mock phosphorylation reaction was diluted into polymerization buffer to a final concentration of 2 μM tau as above, except that there was no carry over of GSK-3β or of ATP if those reagents were omitted from the mock phosphorylation reaction.

#### ThS containing reactions

Polymerization reactions with ThS contained 2 μM non-phosphorylated tau in buffer containing 10 mM HEPES, pH 7.64, 100 mM NaCl, 0.1 mM EDTA, 5 mM DTT and 20 μM ThS. ARA was added to a final concentration of 25 or 75 μM to induce polymerization. Reactions with phosphorylated or mock phosphorylated tau resulted in minor buffer additions (described above). ThS containing reactions were used in Figure 3 to compare levels of polymer for the kinetics study and also for Figures 4, and 6, 7, 8, 9.

### Kinetics

The kinetics of polymerization reactions were assayed by ThS fluorescence, utilizing a FlexStation II fluorometer microplate reader (Molecular Devices Corporation, Sunnyvale, CA). Settings included: Excitation λ = 440, Emission λ = 520, PMT = high. Polymerization reactions containing all reagents except ARA were prepared. The FlexStation II automatically added the ARA and began fluorescence intensity readings thirteen seconds later. Readings were taken every 1.5 seconds for the first 30 minutes, then once every 5 minutes for 20 hours.

### Electron microscopy of tau filaments and filament clusters

#### Grid preparation

Samples were diluted either by a factor of five (25 μM ARA reactions) or ten (75 μM ARA reactions) in polymerization buffer, then applied to the grids, allowing one minute for filaments and/or clusters to attach. The edge of the grid was then touched to filter paper to blot away excess liquid. Grids were stained with 1% uranyl acetate for one minute then blotted as above. Grids were viewed with a JEOL 1200 EXII electron microscope and images were captured with the MegaViewII imaging system (Soft Imaging System, GmbH Münster, Germany).

#### Measurements of tau filaments and filament clusters

The area, mass and density of localized accumulations of tau filaments on electron microscopy grids were measured using the Optimas analytical imaging software (Media Cybernetics, Silver Spring, MD) and GraphPad Prism software (Graphpad Software, San Diego, CA). Digital electron micrographs were collected at 20,000 × magnification. For measurements of individual filaments, the entire field was selected using the "threshold tool" and "auto find lines" feature of the software. For clusters of filaments, the region of interest tool was used to outline the clusters of filaments in Optimas. Filaments within the clusters were selected using the "threshold tool" and the "auto find lines". For a field of individual filaments or for filaments in the selected "area of interest" cluster, the number and average length of filaments was determined and multiplied to obtain an estimate of filament mass in the field or region of interest. For clusters, the "draw area tool" was used to outline the outer boundary of the filaments in order to obtain the area occupied. The total sum length of filaments was divided by the area occupied by those filaments to obtain measurements for the density of the filament clusters.

### Discontinuous Sucrose Gradients

Centrifugation of tau polymerization reactions through discontinuous gradients consisting of 1, 1.5 and 2 M sucrose were performed as previously described [33], except that sucrose was dissolved in buffer containing 5 mM DTT, 100 mM NaCl, 10 mM HEPES, and 0.1 mM EDTA. Polymerization reactions were overlaid on the gradient and centrifuged at 100,000 × g for 2 h in a TLA 100.3 rotor (Beckman-Coulter, Fullerton, CA). To compensate for any reduction of filament adherence due to sucrose in the samples, gradient fraction samples were applied 5 times to the formvar carbon coated electron microscopy grids, allowing 1 minute each time for filaments to attach. Grids were rinsed five times with polymerization buffer and stained with 0.5% uranyl acetate for 1 minute. Therefore, the filaments viewed by electron microscopy were concentrated five fold in addition to any concentration of filaments produced by the centrifugation through the gradient.

## List of abbreviations

AD Alzheimer's disease

ARA arachidonic acid

ThS thioflavine S

GSK-3β glycogen synthase kinase 3β

NFT neurofibrillary tangle

TEM transmission electron microscopy

## Competing interests

The author(s) declare that they have no competing interests.

## Authors' contributions

CAR participated in the design of the study, performed the phosphorylation reactions, SDS-PAGE analysis, phosphate incorporation, phosphorylation dot blots, polymerization reactions, electron microscopy and drafted the manuscript. QS performed the kinetic analysis of polymerization. TCG conceived of the study, and participated in its design and coordination and helped to draft the manuscript. All authors read and approved the final manuscript.
